# Heterogeneous Thermochromic Hydrogel Film Based on Photonic Nanochains

**DOI:** 10.3390/nano12111867

**Published:** 2022-05-30

**Authors:** Hexuan Yan, Luying Si, Gang Li, Lejian Zhao, Wei Luo, Huiru Ma, Jianguo Guan

**Affiliations:** 1 State Key Laboratory of Advanced Technology for Materials Synthesis and Processing, School of Materials Science and Engineering, Wuhan University of Technology, Wuhan 430070, China; yanhx@whut.edu.cn (H.Y.); chemligang2010@whut.edu.cn (G.L.); 2 State Key Laboratory of Advanced Technology for Materials Synthesis and Processing, International School of Materials Science and Engineering, Wuhan University of Technology, Wuhan 430070, China; siluying@whut.edu.cn (L.S.); 298552@whut.edu.cn (L.Z.); guanjg@whut.edu.cn (J.G.); 3 School of Chemistry, Chemical Engineering and Life Science, Wuhan University of Technology, Wuhan 430070, China

**Keywords:** thermochromic film, heterogeneous hydrogel, photonic nanochains, quick response, large deformation

## Abstract

The rapid and robust response to external stimulus with a large volume deformation is of huge importance for the practical application of thermo-responsive photonic crystal film (TRPCF) in actuators, colorimetric sensors, and other color-related fields. Generally, decreasing the size of thermo-responsive photonic crystals and introducing micropores are considered to be two effective approaches to improve their responsiveness. However, they usually result in a poor mechanical property, which leads to optical instability. To solve these problems, a heterogeneous thermo-responsive photonic crystal film was developed here by integrating a thermosensitive hydrogel matrix poly(*N*-isopropylacrylamide-co-*N*-methylolacrylamide) (P(NIPAM-co-NHMA)) with high-modulus, but non-thermosensitive poly(acrylic acid -co-2-hydroxyethyl methacrylate (P(AA-co-HEMA)) hydrogel-based photonic nanochains (PNCs). The as-obtained TRPCF based on PNCs (TRPCF-PNC) well combined the rapid response and improved the mechanical property. Typically, it can complete a response within 12 s from 26 to 44 °C, which was accompanied by a larger deformation of the matrix than that of the PNCs. The unique rapid thermochromic mechanism of the TRPCF-PNC is revealed here. Furthermore, it exhibits a high tensible property along the PNC-orientation direction and excellent optical stability. The response time of the TRPCF-PNC can conveniently modulate by changing the cross-linking degree of the PNCs or the content of the thermosensitive component in the matrix. The heterogeneous TRPCF-PNC is believed to have potential applications in artificial muscle and quick-response actuation devices.

## 1. Introduction

Thermosensitive smart materials have vigorous development in frontier research fields such as thermoresponsive grafted brush coatings [1], biomedical applications [2], biomolecule sensing systems [3], soft actuators [4], smart windows [5], and so on. Of these materials, hydrogel-based thermally responsive photonic crystal films (TRPCFs) have attracted increasing attention because of their promising applications in flat-panel displays, optical switches, sensors, and temperature monitoring [6,7,8,9,10]. Various types of TRPCF including thermosensitive hydrogel-based opals [11], inverse opals [12,13], Bragg stacks [14,15], magnetically responsive photonic crystals [16,17], etc., have been reported, and they intuitively exhibit color transformation as well as reflection spectra shift in response to ambient temperature change [17,18]. Generally, they are composed of homogeneously responsive hydrogel and photonic crystals, and thus the optical response of hydrogel-based photonic crystals is directly correlated with the volumetric changes in the hydrogel matrix. The maximum of the reflection peak is dictated by the lattice spacing (d) of the photonic crystals via Bragg’s law, and d is proportional to the heating induced changes in the hydrogel volume [19,20].

However, TRPCFs usually suffer from weak mechanical strength, sluggish response speed, and the distortion of periodic structures, limiting their real-world applications [21]. According to the dynamic model of gel proposed by Tanaka and Fillmore, the relaxation time of the gel is proportional to the square of the gel size and inversely proportional to its collective diffusion coefficient [22]. Following this theory, various works have been designed to address the above challenges in the past decades [23,24,25]. For example, a TRPCF with an inverse opal structure was fabricated by first utilizing colloidal particles to form an opal template and infiltrating poly(*N*-isopropyl acrylamide) (PNIPAM). After etching away the SiO_2_ opal templates, the remaining interconnected porous inverse opal could exhibit color changes depending on the temperature. Although the response rate had been accelerated, the porosity further deteriorated the mechanical strength of the hydrogel and made a photonic crystal structure fragile [26]. In another method, a rapidly modulated TRPCF was prepared via the assembly of polystyrene@poly(*N*-isopropylacrylamide-co-bisacrylamide-co-acrylic acid, core-shell particles. The rapid volume change caused by the thin poly(*N*-isopropylacrylamide-co-bisacrylamide-co-acrylic acid hydrogel shell contributed to the high response speed [27]. Unfortunately, the TRPCF obtained by close-packed particles also possessed weak mechanical properties. In order to improve the structure stability, an intact self-supporting TRPCF was constructed by cross-linking the shell of the colloidal particles after the assembly of 3D opal structures, and an instantaneous color adjustment was achieved by heat stimuli [28]. Nevertheless, the high-volume content of the colloidal particles in the opal structure coupled with the easily generated defects make TRPCFs appear vulnerable in the structures and non-uniform in color.

In this paper, a heterogeneous TRPCF was proposed by choosing non-temperature responsive peapod-like photonic nanochains (PNCs) as the basic color display units and embedding them in a poly(*N*-isopropylacrylamide-co-*N*-methylolacrylamide) (P(NIPAM-co-NHMA)) hydrogel matrix (TRPCF-PNC) via an external magnetic field-assisted in situ polymerization triggered by UV light. The hydrogel shell of the PNCs is composed of poly(acrylic acid-co-2-hydroxyethyl methacrylate (P(AA-co-HEMA)), which is insusceptible to the temperature change and totally different to the component of the hydrogel matrix. The microstructure and composition of TRPCF-PNC were characterized using scanning electron microscopy (SEM), optical microscopy, and Fourier transformed infrared (FTIR), respectively. In addition, the thermochromic mechanism of the heterogeneous TRPCF-PNC and the influence factors of response time were investigated via a fiber optic spectrometer. The heterogeneous TRPCF-PNCs not only pave a way for developing a novel TRPCF, but also show promising potential in extensive fields such as actuators, wearable electronics, and sensors.

## 2. Materials and Methods

### 2.1. Materials

Acrylic acid (AA, 99%), 2-hydroxy-2-methylpropiophenone (HMPP, 97%), 2-hydroxyethyl methacrylate (HEMA, 99%), ethylene glycol dimethacrylate (EGDMA, 98%), *N*-isopropylacrylamide (NIPAM, 98%), *N*-methylolacrylamide (NHMA, 98%), *N,N′*-methylenebisacrylamide (BIS, 100%), and 3-(trimethoxysilyl)propyl methacrylate (KH570, 97%) were purchased from Aladdin. Ethanol (EG) (AR, ≥99.7%) and ethylene glycol (AR, ≥99.5%) were provided by Sinopharm Chemical Reagent Co. Ltd. (Shanghai, China). All chemicals were used directly as received without further purification. Deionized water was processed by a MilliQ Advantage. The used superparamagnetic magnetite@polyvinylpyrrolidone (Fe_3_O_4_@PVP) colloidal nanocrystal clusters were prepared by our previously reported method [29].

### 2.2. Preparation of Fe_3_O_4_@PVP @P(AA-co-HEMA) PNCs

The Fe_3_O_4_@PVP@P(AA-co-HEMA) PNCs were prepared following our previously reported method [30]. For a typical process, 1.5 mg of Fe_3_O_4_@PVP with a uniform diameter of about 180 nm, 0.2541 g AA, 0.2082 g HEMA, 0.0014 g HMPP, and 0.0256 g EGDMA were added in a 25 mL beaker containing a 1523 μL solution of 1323 μL EG and 200 μL deionized water, and ultrasonically dispersed to form a homogeneous precursor solution. Afterward, the beaker was placed above a neodymium (NdFeB) permanent magnet to provide a 500 Gauss magnetic field. The Fe_3_O_4_@PVP nanoparticles in the precursor solution were magnetically induced to assemble into chain-like photonic crystal templates, resulting in a bright green before the polymerization. Two minutes later, in situ template polymerization was carried out under magnetic field-assisted UV irritation for 5 min. Finally, the product was washed with ethanol 2~3 times.

Moreover, PNCs with a cross-linking degree of 4% and 6% were prepared according to the above experimental process by just varying the cross-linker (EGDMA) content to 0.0128 g and 0.0192 g, respectively. All of the samples were preserved in ethanol before use.

### 2.3. Fabrication of Heterogeneous TRPCF Composed of Fe_3_O_4_@PVP@P(AA-co-HEMA) PNCs and P(NIPAM-co-NHMA) Matrix

The heterogeneous thermochromic photonic crystal films were fabricated on a double bond modified glass substrate through external magnetic field-assisted in situ UV polymerization. A piece of glass substrate was prepared in advance, which was carefully treated by piranha solution and modified by KH570. Furthermore, a 100 μm-thickness round gasket cavity (Φ = 2 cm) as a mold was covered on the modified glass substrate. Next, in a typical process, NIPAM (0.16 g), NHMA (0.04 g), BIS (0.005 g), and HMPP (4 μL) were added orderly into a 600 µL ethanol and deionized water (1:1) solution. A total of 2 mg of the prepared PNCs from a typical experiment was dispersed into the mixed solution and stirred until a homogeneous dispersion was formed before being kept in a dark place. Afterward, 100 μL of the above precursor solution was quickly put into the round gasket cavity and a piece of cover glass was placed on it to form a closed chamber, under which a NdFeB permanent magnet was placed to provide an approximate 500 Gs external magnetic field to produce the PNCs’ rotational orientation. Two minutes later, UV light was applied to trigger an external magnetic field-assisted in situ polymerization, and orientated Fe_3_O_4_@PVP@P(AA-co-HEMA) PNCs were embedded into the P(NIPAM-co-NHMA) matrix. In such a way, a heterogeneous TRPCF-PNC composed of Fe_3_O_4_@PVP@P(AA-co-HEMA) PNCs and a P(NIPAM-co-NHMA) matrix was firmly anchored on the glass substrate and obtained by peeling off the cover glass and gasket cavity, then washing it with deionized water five times to remove the unreacted substances including ethanol, before finally immersing it into deionized water. 

Heterogeneous TRPCF-PNC with different compositions were obtained by using the above fabrication method but altering the copolymer monomer ratio of NIPAM and NHMA or by using Fe_3_O_4_@PVP@P(AA-co-HEMA) PNCs with different crosslinking degrees. All of the resultant hydrogel film samples were immersed into water overnight to reach the equilibrium swelling state at 26 °C before use.

### 2.4. Characterizations

All digital photos in this paper were taken using a Huawei P30 mobile phone. The microstructures of the PNCs and TRPCF-PNC were investigated by a Hitachi S-4800 SEM and optical microscope (Zeiss Axio Observer 5M, Göttingen, Germany). The FTIR spectra were obtained using a Nicolet 6700 FTIR spectrometer in the range of 400–4000 cm^−1^ with a resolution of 4 cm^−1^. A fiber optic spectrometer (Ocean Optics USB 2000+) was used to record the reflective spectra in the range of 400–1000 cm^−1^. The testing temperature was controlled through a water bath and the response time was recorded using a Huawei P30 mobile phone timing app. The bar-type sample was prepared and stretched via two tweezers from both ends. The initial length of the sample between the tweezers was recorded as L_0_, and the stretched length at the beginning of the fracture was recorded as L_a_. The elongation at break was calculated according to the formula: e = (L_a_ − L_0_)/L_0_.

## 3. Results

**Figure 1:**
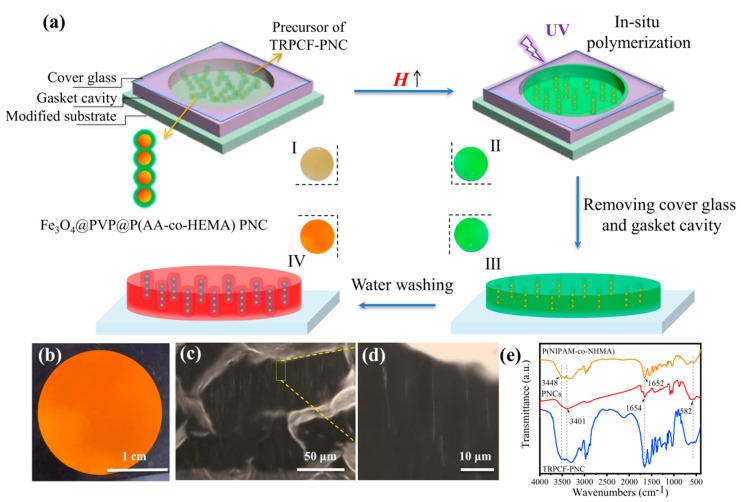
(**a**) A schematic diagram of the preparation process of the thermo-responsive photonic crystal film based photonic nanochains (TRPCF-PNC). First, a precursor solution containing photonic nanochains (PNCs) was injected into the gasket cavity clapped between a double-bond modified glass substrate and a cover glass. Then, the whole sample was placed in a uniform magnetic field to induce the alignment of PNCs, followed by UV curing. The TRPCF-PNC was eventually obtained after removing the cover glass and gasket cavity, which were then washed. The digital photos of the four states of the TRPCF-PNC in different procedures of preparation are presented correspondingly (I to IV). (**b**) Digital photo, (**c**) cross-section, and (**d**) locally magnified optical microscopy images of typical TRPCF-PNC. (**e**) FTIR spectra of the different samples.

Unlike the conventional TRPCFs, a new preparation strategy is proposed here to fabricate a heterogeneous TRPCF-PNC in which Fe_3_O_4_@PVP@P(AA-co-HEMA) PNCs are used as display units. Figure 1a schematically shows the preparation process of the TRPCF-PNC as well as the digital photos corresponding to the different stages (I to IV) of the preparation process. The Fe_3_O_4_@PVP@P(AA-co-HEMA) PNCs were first fabricated according to the reported strategy [30]. The SEM image of the PNCs in Figure S1a illustrates that the colloidal particles that had an equal interparticle spacing were coated by the gel, presenting a peapod-like structure. The optical microscope images in Figure S1b,c demonstrate that the PNCs were relatively rigid, which is consistent with previous analogs [31]. Furthermore, as shown in Figure S2, the reflection spectra of these PNCs are independent of the temperature change, proving their non-temperature responsiveness. In the meantime, once aligned with the direction of the external magnetic field, the reflection peak position of the PNCs as well as their reflective intensity are almost immune to the increment in the magnetic field strength (Figure S3). During the TRPCF-PNC preparation process, a precursor solution is injected into the gasket cavity loaded onto a carbon–carbon double bond modified glass substrate and the whole sample shows a brown color (photo I). When a vertical magnetic field is applied normally to the glass substrate, the sample appears green due to the rapid alignment of PNCs along the magnetic field (photo II). Next, in situ polymerization is initiated by ultraviolet radiation, and those oriented PNCs are fixed by the surrounding thermosensitive hydrogel. The prepared TRPCF-PNC is covalently anchored on the substrate via the chemical reaction between the monomers and silane-modified glass substrate. Generally, the contraction of volume will occur when monomers transform into macromolecules, which may result in the blue shift of the embedded non-closed packed photonic crystals. However, the TRPCF-PNC still maintained the same green (III) as that of its precursor solution, which can be attributed to the prefixed periodic structure of the PNCs and shows that PNCs have sufficient mechanical strength to resist the internal stress generated during polymerization. After removing the cover glass and gasket cavity and immersing the TRPCF-PNC into deionized water to reach a swelling balance, the structural color of the rinsed TRPCF-PNC exhibited red (IV) and the reflection spectrum also red shifted (Figure S4). This phenomenon implies that after the incorporation of PNCs into the thermo-responsive matrix, the PNCs acquire thermal responsiveness. 

Thanks to the confinement effect from the substrate, the surface of the as-obtained typical TRPCF-PNC can be kept flat and smooth at near ambient temperature (Figure 1b). The cross-section of TRPCF-PNC can be observed in Figure 1c via optical microscope, where there many one-dimensional oriented PNCs were distributed. No type of PNC aggregates was found in the locally magnified photograph in Figure 1d, indicating good compatibility between the PNCs and the P(NIPAM-co-NHMA) hydrogel matrix. The SEM images show the clear pore structure of the gel and the ordered photonic crystal chains can be observed (Figure S5). The compositions of the Fe_3_O_4_@PVP@P(AA-co-HEMA) PNCs, P(NIPAM-co-NHMA), and typical TRPCF-PNC were characterized by the FTIR spectra in Figure 1e. For the curve of the PNCs, the absorption peaks at the peaks located at 1654 cm^−1^ and the broad absorption peak that started at 3401 cm^−1^ were due to the -COOH of AA. In addition, the sharp absorption peak at 582 cm^−1^ was due to the stretching vibration absorption of the Fe–O in Fe_3_O_4_ [24]. The weak organic absorption peak indicates a lower gel content of the PNCs. By analyzing the P(NIPAM-co-NHMA) curve, the two moderate stretching vibration absorption peaks at 3448 cm^−1^ and 1652 cm^−1^ were due to the N–H and C=O of the amide group [32]. The above peaks all appeared in the curve for TRPCF-PNC, which proved the composition of the composite film.

**Figure 2:**
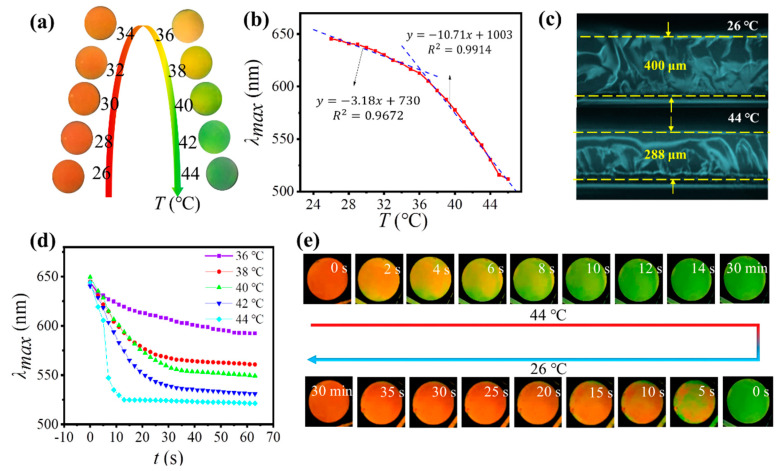
(**a**) Digital photos of the TRPCF-PNC under different temperatures in the range of 26 °C~44 °C. (**b**) The maximum peak wavelength (λ_max_) as a function of temperature (**c**) Cross-sectional optical microscope images of the TRPCF-PNC under 26 °C and 44 °C, respectively, showing large deformability along its thickness direction. (**d**) λ_max_ as a function of *t* when the same film is transferred from 26 °C to different water baths with various temperatures. (**e**) A series of digital photos recorded a recycled color change of TRPCF-PNC when the film was first transferred from a water bath of 26 °C to 44 °C and then back to 26 °C.

As expected, though the original Fe_3_O_4_@PVP@P(AA-co-HEMA) PNCs were not sensitive to heating, the TRPCF-PNC exhibited a distinctive and rapid optical change behavior in response to temperatures ranging from 26 °C~44 °C. A series of digital photos in Figure 2a reveals that the TRPCF almost stayed red and displayed a trivial blue shift inf structural color when the temperature rose from 26 °C to 34 °C. Subsequently, the structural color varied obviously from brilliant orange to green, induced by the temperature change from 34 °C to 44 °C. Correspondingly, the curve for the maximum peak wavelength (λ_max_) of the TRPCF-PNC as a function of temperature in Figure 2b displayed two areas with distinctly different slopes between 26~44 °C with the transition point located at 34 °C. This phenomenon is unique as traditional homogeneous TRPCFs (TRPCF-P), which only contain 1D aligned chain-like nanoparticles buried in a pure thermal-responsive hydrogel, linearly shift their λ_max_ in the same temperature range (Figure S6) [16,17]. In addition, the thermal tunable range of TRPCF-P covers a wider visible spectrum than that of TRPCF-PNC.

Furthermore, Figure 2c shows the cross-section of the TRPCF-PNC under 26 °C and 44 °C. The ratio between the film thicknesses at these two different states reached 1.39. Since the film is covalently attached on the glass substrate via silane, the volume deformation of the TRPCF-PNC is not isotropic, but limited to the direction perpendicular to the substrate and converted into the changes in thickness. It is well-known that for a traditional homogeneous TRPCF, the change in thickness is proportional to the change in the lattice constant normal to the substrate of the photonic crystal embedded in the hydrogel. The homogeneous TRPCF-P in Figure S5 also proved this conclusion because the ratio of λ_max_ was 1.38 when exposed to the same stimuli and almost equal to the thickness change ratio of the P(NIPAM-co-NHMA) matrix (1.39). It suggests that for TRPCF-P, the change in the interparticle distance can fully follow the shrinkage/swelling of the matrix as they had the same hydrogel composition. However, the ratio of the different λ_max_ of the TRPCF-PNC corresponding to 26 °C and 44 °C was about 1.22 and far below the above expansion ratio (1.39) of the P(NIPAM-co-NHMA) hydrogel matrix. The differences revealed a different tuning principle on the photonic bandgaps under the thermal stimuli between the TRPCF-PNC and TRPCF-P.

The fast responsive rate to external stimulus has been a critical theme for the practical application of responsive photonic crystals. Fortunately, what distinguishes the as-prepared heterogeneous TRPCF-PNC from the previous homogeneous ones is that it can present an instant response to temperature change with a large volume deformation. Different response times were measured by monitoring the λ_max_ when the TRPCF-PNC was transferred from 26 °C to 36 °C, 38 °C, 40 °C, 42 °C, and 44 °C, respectively. The response time was defined as the time the TRPCF-PNC needed to reach 90% of the reflection wavelength shifts from 26 °C to those higher temperatures. As depicted in Figure 2d, the λ_max_ of the TRPCF-PNC could all balance within 1 min, demonstrating the rapid response of the TRPCF-PNC to the temperature. In particular, it only took 12 s for the TRPCF-PNC to become stable at 44 °C. All of the color change processes were recorded by video (Video S1) and the digital photos in Figure 2e. In comparison, TRPCF-P showed a much longer response time in Figure S7.

**Figure 3:**
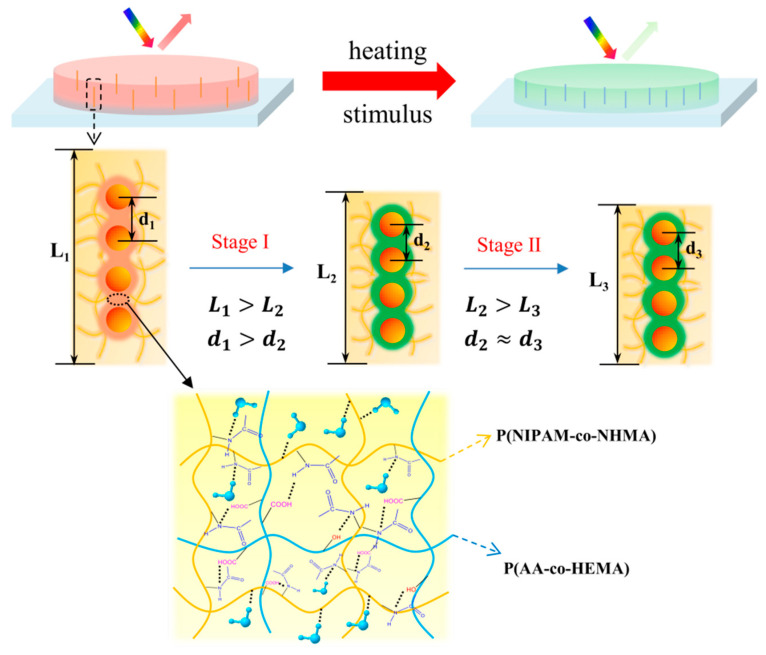
A schematic illustration of the thermochromic mechanism of TRPCF-PNC: The P(NIPAM-co-NHMA) hydrogel matrix had a larger deformation in the thickness direction than the embedded PNCs. The magnified area shows that the interpenetrating network formed in the hydrogel of the PNCs after polymerization, which endowed them with a thermos-responsiveness to the PNCs.

In order to clarify the distinctive and rapid response behavior of TRPCF-PNC to the temperature change, a possible thermochromic mechanism for TRPCF-PNC was proposed and the schematic model is depicted in Figure 3. When the precursor solution containing NIPAM, NHMA, ethanol, and water is first mixed with PNCs, monomers will infiltrate into the P(HEMA-co-AA) shell of the PNCs. This is because the mixture of ethanol and water is a good solvent to swell the P(HEMA-co-AA), which might bring monomers into the hydrogel shell. Thus, after polymerization, they form an interpenetrating network in the hydrogel shell of PNCs, which endows them with thermal responsiveness to the PNCs. Since the shell has a high cross-linking degree and is more rigid than the matrix, the temperature related color tunability is low when compared to the TRPCF-P. Furthermore, according to the literature, the more hydrophilic the copolymer relative to PNIPAM, for example, PAA in the polymer shell will shift the phase transition point of PNIPAM to higher temperatures [33,34]. Thus, the two different slopes of TRPCF-PNC in Figure 2b intersect around 34 °C rather than at 28 °C of TRPCF-P. In order to further prove the above speculation, a control experiment was implemented in Figure S8 where the P(HEMA-co-AA) of the PNCs were replaced by pure PHEMA. Due to the lack of hydrophilic PAA, the same intersection point of the TRPCF-PNC integrated with PNCs@PHEMA changed back to 28 °C, which is consistent with our expectation.

The fast response rate of the TRPCF-PNC than that of the TRPCF-P could be explained by the high cross-linking degree of the PNCs for the following two reasons. First, hydrogels with greater rigidity tend to respond quickly for their higher modulus [35,36]. Second, the high modulus polymer between the nanoparticles of the PNCs impedes them from being further compressed, even if the hydrogel matrix is still in its deswelling process when the temperature is high. As shown in Figure 3, the PNCs first contracted following the tendency of the volume deformation of the hydrogel matrix (stage I, L_1_ > L_2_, d_1_ > d_2_), but they reached the equilibrium state faster than that of the hydrogel matrix (stage II, L_2_ > L_3_, d_2_ ≈ d_3_) due to the existence of a high modulus polymer resistance to further compression.

**Figure 4:**
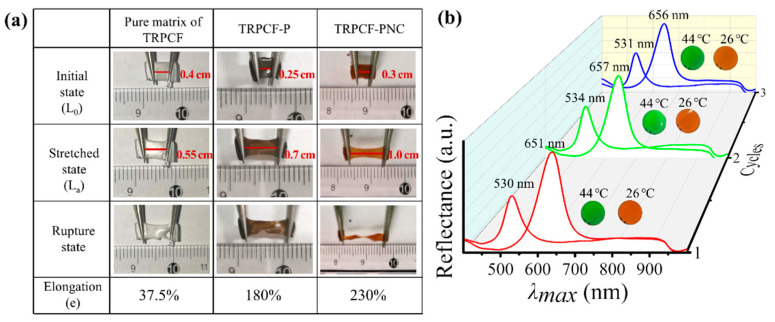
(**a**) The mechanical properties of the pure matrix of TRPCF, TRPCF-P, and TRPCF-PNC with a thickness of 1 cm were tested by using tweezers to stretch. (**b**) The cycling stability of TRPCF was obtained by monitoring the reflection spectrum and structural color variation through repeatedly switching the sample from 26 °C to 44 °C every two months.

Though responsive photonic crystal films can undergo a large volume change and present an obvious variation in optical properties to external stimulus, the poor mechanical performance severely affects their optical stability, thus limiting their practical application. Here, the mechanical performance of a typical TRPCF-PNC was investigated and was expected to be improved by the embedded Fe_3_O_4_@PVP@P(AA-co-HEMA) PNCs. For this purpose, a sample with the same formula but horizontally oriented PNCs was prepared to guarantee the consistence of the stretched direction of the P(NIPAM-co-NHMA) matrix and the alignment direction of the PNCs. Furthermore, TRPCF-P and pure P(NIPAM-co-NHMA) matrix samples to test the mechanical performance as contrast samples were also prepared. As shown in Figure 4a, the initial state of all of the samples was smooth and compact. The TRPCF-PNC presented the maximum elongation at break of 230%, which was nearly six times and 1.3 times of that of the elongation at break of the pure P(NIPAM-co-NHMA) matrix and the TRPCF-P samples, respectively. This proves that the chain-like PNCs, just like multi-walled carbon nanotubes [37] and cellulose nanocrystalline [38,39], play a significant role in absorbing energy as well as resisting crack propagation during the deformation process, thus enhancing the toughness and strength of the TRPCF-PNC sample.

Benefitting from the good toughness in the direction of the volume deformation, TRPCF-PNC shows good cycling stability, which is another crucial aspect for the consideration of its practical application, as shown in Figure 4b. Accordingly, three cycle experiments of the reflection spectrum and structural color versus temperature were carried out by repeatedly switching the typical TRPCF-PNC sample from 26 °C to 44 °C every two months. The test results are presented in Figure 4b, which indicates that both the reflection peak intensity and position of TRPCF-PNC almost remained unchanged at 26 °C and 44 °C after experiencing three cycles during six months. Additionally, obvious fading of the structural color could not be observed in the TRPCF-PNC during the thermochromic process due to the prefixed ordered structures in PNCs [40,41,42]. The excellent optical stability and durability of TRPCF-PNC were attributed to its unique heterogeneous structure, which endowed it with robust and reversible volume deformation along the PNCs’ orientation direction.

**Figure 5:**
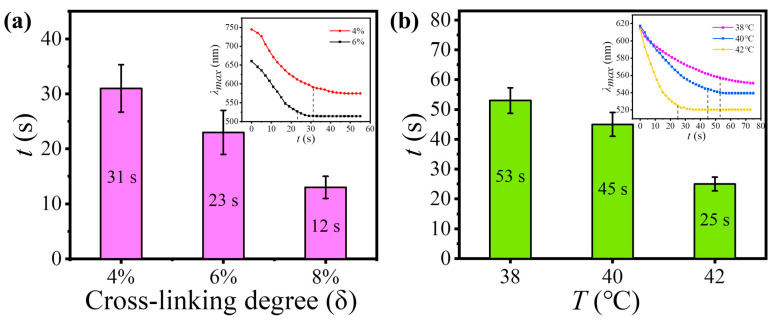
(**a**) The response time of TRPCF-PNC as a function of the cross-linking degree of the embedded PNCs. The inset shows the shift in the reflection wavelength at different points in time when TRPCF-PNC was transferred to 44 °C from 26 °C. (**b**) The response time graph under different stimulus temperatures of TRPCF-PNC with 10 wt% NHMA in the P(NIPAM-co-NHMA) matrix. The insert shows the corresponding change curves of the reflection wavelength with time during the stimulus process.

The response time of TRPCF-PNC to thermal stimulus can be conveniently tuned by employing PNCs with different mechanical strength through the change in the cross-linking degree (δ), but without varying the composition of the P(NIPAM-co-NHMA) matrix. Figure 5a gives the response times of TRPCF-PNC with different δ of the embedded PNCs when the samples were transferred from 26 °C to 44 °C. It shows that a higher δ of the PNCs produced a faster response rate for TRPCF-PNC, which is in accordance with the aforementioned mechanism. The response time of TRPCF-PNC was shorted by 2.5 times when δ increased from 4% to 8%. The curves of λ_max_ with time in the inset, together with Figure 2d, show the cross-linking degree of the PNCs on the response time of TRPCF-PNC to thermal actuation. It proves that the PNCs at a high cross-linking degree have a stronger ability to resist the deformation from the matrix. Over time, it will become so strong in a shorter time, that the continuous shrinkage of the P(NIPAM-co-NHMA) matrix is unable to further narrow the interparticle space of the PNCs. As a result, the TRPCF embedded with PNCs at a higher cross-linking degree can exhibit a faster response to external stimulus.

The effect of the P(NIPAM-co-NHMA) hydrogel matrix on the response time of TRPCF-PNC was also explored by decreasing the NHMA content from the typical 20 wt% to 10 wt%. Compared with the typical TRPCF-PNC, the dependency relationship between the response time and temperature stimulus in Figure 5b was obviously slower. Furthermore, according to the inset in Figure 5b, one can see that its original reflection peak position was smaller than that of a typical sample, demonstrating a weaker driving force from the P(NIPAM-co-NHMA) hydrogel matrix. As a result, upon external stimulus, the P(NIPAM-co-NHMA) hydrogel matrix was unable to exert a sufficient strong acting force via volume shrinkage to make the PNCs generate a large and rapid shape change. Just as shown in Figure 5b, compared with a typical sample, it showed a longer response time and its λ_max_ exhibited a narrower shift range under the same stimulus temperature.

## 4. Conclusions

In summary, we developed a novel PNC-based TRPCF that has the ability to combine a rapid response with a large volume deformation of a thermosensitive matrix. The key technology lies in its heterogeneous hydrogel composition between the matrix and PNCs as well as a higher modulus of PNCs than the hydrogel matrix. Typically, the PNC-based TRPCF can instantly change the structural color and achieve an optical stability within 12 s when being applied at a 44 °C temperature actuation. An unconventional rapid thermochromic mechanism discovered that the thermo-induced volume deformation of the matrix caused a relatively small deformation of the PNCs, which shortens the response time of the TRPCF during a long-time deformation process of the matrix. Additionally, the embedded PNCs enhanced the mechanical property of the TRPCF and rendered it with excellent optical stability in the cycle experiment. Furthermore, the response time of the TRPCF can be conveniently modulated by employing PNCs with different cross-linking degrees or adjusting the composition of the thermosensitive matrix. Therefore, we believe that the PNC-based TRPCF reported here will have an important prospect in artificial muscle and quick-response actuator devices.

## Data Availability

The data presented in this study are available on request from the corresponding author.

## Supplementary Materials

The following supporting information can be downloaded at: https://www.mdpi.com/article/10.3390/nano12111867/s1, Figure S1: The SEM and optical microscopy images of the Fe_3_O_4_@PVP@P(AA-co-HEMA) PNCs; Figure S2: The reflection spectrum of the PNCs at different temperatures; Figure S3: The reflection spectrum of the PNCs under different magnetic fields; Figure S4: The reflection spectrum of the PNCs and the swelling-equilibrated TRPCF-PNC; Figure S5: The SEM images of the TRPCF-PNC and magnification of one PNC; Figure S6: Th reflection wavelength at the maximum reflection intensity of the TRPCF-P as a function of temperature; Figure S7: The reflection wavelength as a function of *t* when transferring TRPCF-P from a 26 °C water bath to a 44 °C water bath; Figure S8: The reflection peak position curves of the TRPCF prepared with Fe_3_O_4_@PVP@PHEMA PNC as a function of temperature. Video S1: The color change processes of TRPCF-PNC when the film was first transferred from a water bath of 26 °C to 44 °C and then back to 26 °C.
