# Supplementary material for: Heterogeneous Thermochromic Hydrogel Film Based on Photonic Nanochains
Source: Nanomaterials (Basel). 2022 May 30;12(11):1867. doi: 10.3390/nano12111867 (PMC9182477; doi:10.3390/nano12111867)
Supplement: Supplementary file 1 [file nanomaterials-12-01867-s001.zip › nanomaterials-1730621-Supplementary.pdf]

Heterogeneous Thermochromic Hydrogel Film Based on Photonic Nanochains

Hexuan Yan [1], Luying Si [2], Gang Li [1], Lejian Zhao [2], Wei Luo [1,*], Huiru Ma [3,*] and Jianguo Guan [2]

[1] State Key Laboratory of Advanced Technology for Materials Synthesis and Processing, School of Materials Science and Engineering, Wuhan University of Technology, Wuhan 430070, China; yanhx@whut.edu.cn (H.Y.); chemligang2010@whut.edu.cn (G.L.)

[2] State Key Laboratory of Advanced Technology for Materials Synthesis and Processing, International School of Materials Science and Engineering, Wuhan University of Technology, Wuhan 430070, China; siluying@whut.edu.cn (L.S.); 298552@whut.edu.cn (L.Z.); guanjg@whut.edu.cn (J.G.)

[3] School of Chemistry, Chemical Engineering and Life Science, Wuhan University of Technology,
Wuhan 430070, China

* Correspondence: rowell@whut.edu.cn (W.L.); mahr@whut.edu.cn (H.M.)

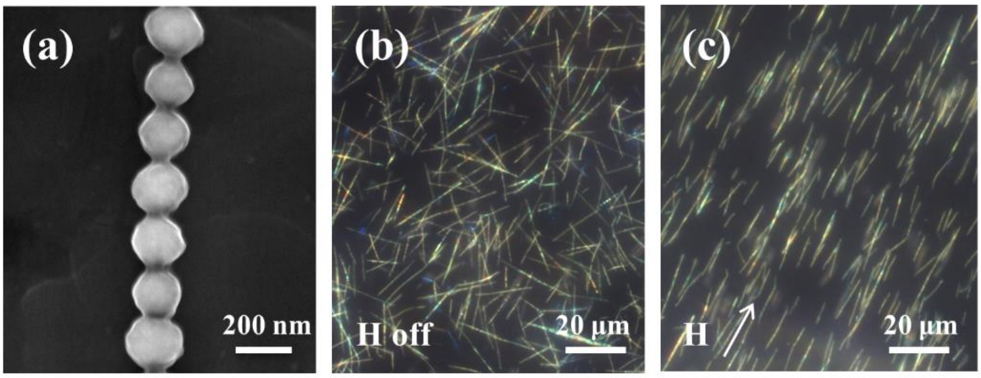

Figure S1. (**a**) SEM of a single Fe_3O_4@PVP@P(AA-co-HEMA) PNC. Dark-field optical microscope images of the same batch of PNCs dispersed in mixed solution of ethanol and water under (**b**) zero, (**c**) a horizontal magnetic field.

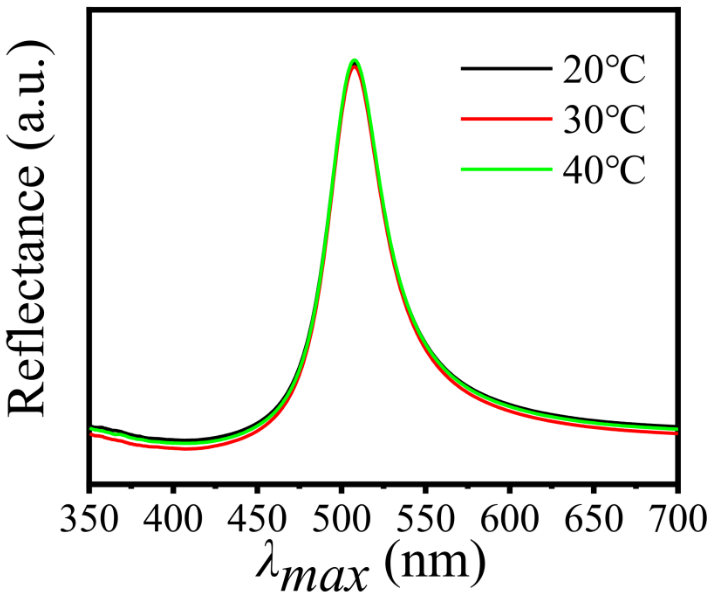

Figure S2. Reflection spectrum of PNCs at different temperature

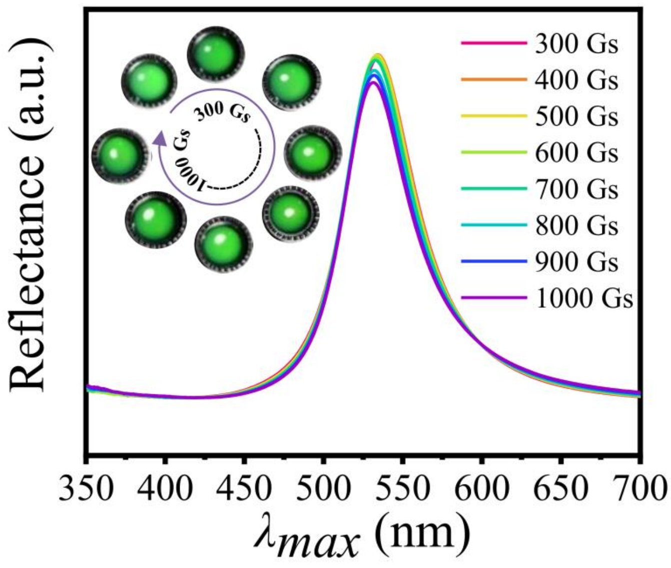

Figure S3. Reflection spectrum of PNCs under different magnetic fields

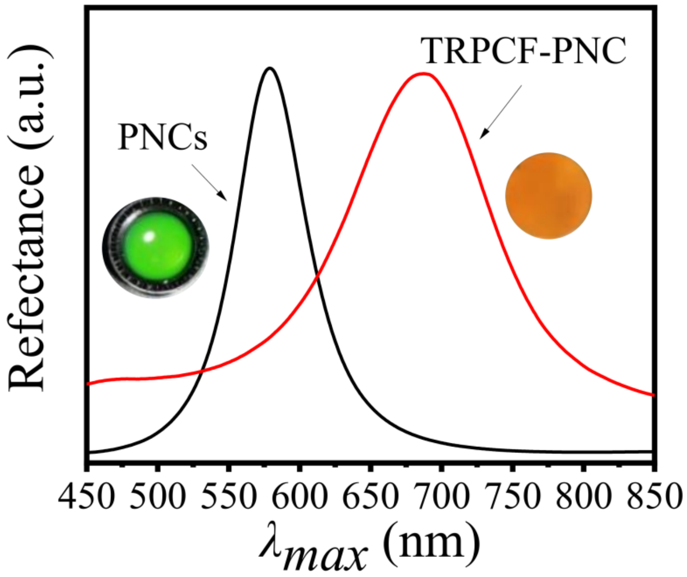

Figure S4. Reflection spectrum of PNCs and swelling-equilibrated TRPCF-PNC

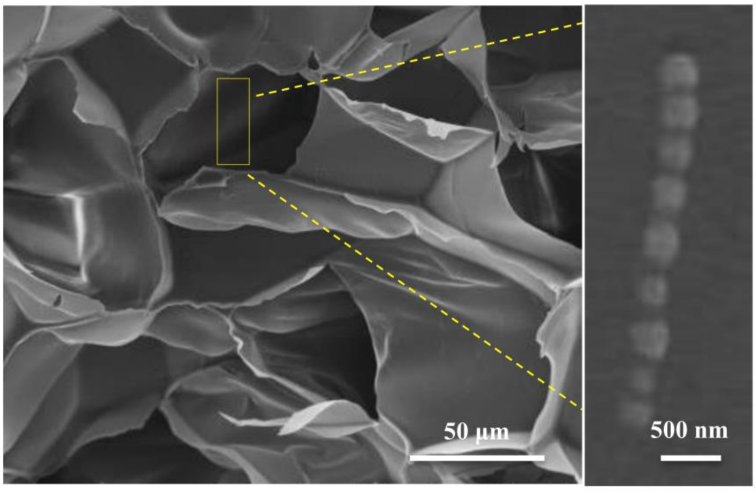

Figure S5. SEM images of TRPCF-PNC and magnification of one PNC.

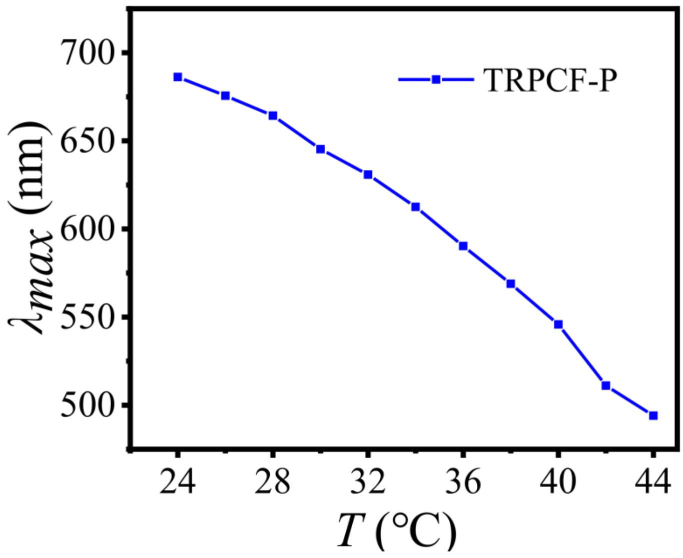

Figure S6. Reflection wavelength at the maximum reflection intensity of TRPCF-P as a function of temperature

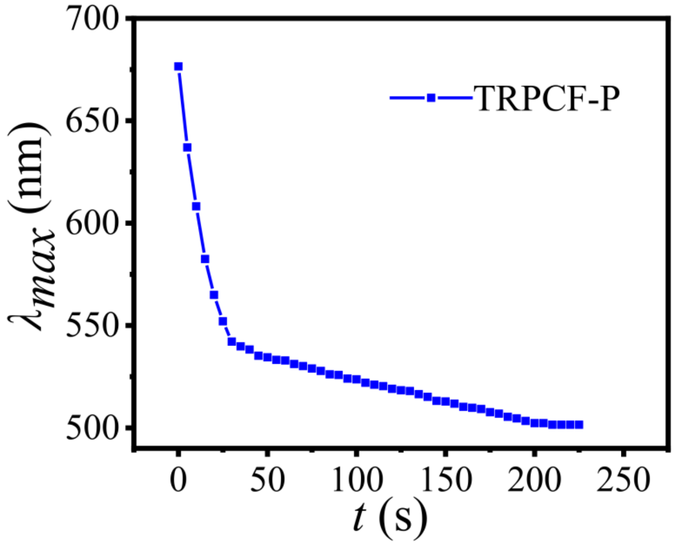

Figure S7. Reflection wavelength as a function of t when transferring TRPCF-P from 26 ^{o}C water bath to 44 ^{o}C water bath

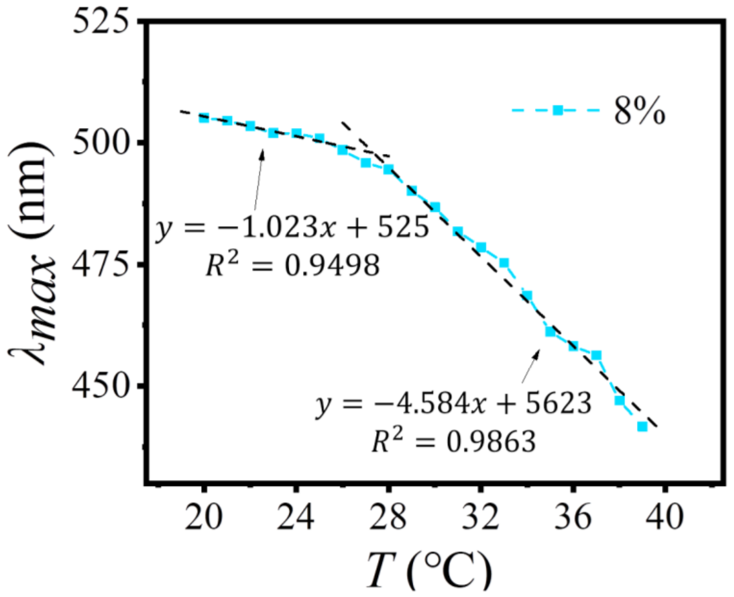

Figure S8. Reflection peak position curves of TRPCF prepared with Fe_3O_4@PVP@PHEMA PNC as a function of temperature
